# Supplementary material for: The Implication of Chemotypic Variation on the Anti-Oxidant and Anti-Cancer Activities of Sutherlandia frutescens (L.) R.Br. (Fabaceae) from Different Geographic Locations
Source: Antioxidants (Basel). 2020 Feb 13;9(2):152. doi: 10.3390/antiox9020152 (PMC7070296; doi:10.3390/antiox9020152)
Supplement: Supplementary file 1 [file antioxidants-09-00152-s001.pdf]

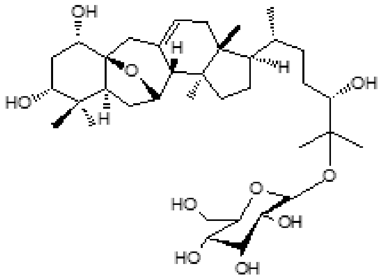

Sutherlandioside A

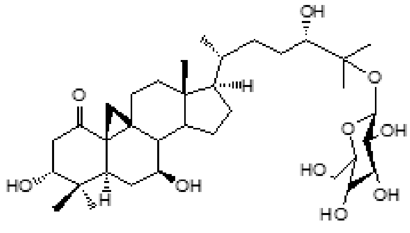

Sutherlandioside B

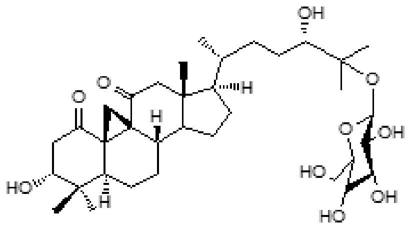

Sutherlandioside C

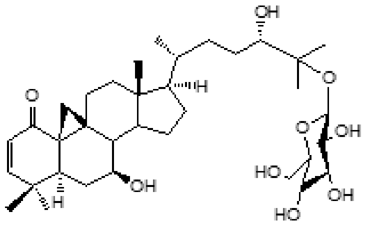

Sutherlandioside D

Figure S1 Structures of terpenoidal saponins (sutherlandiosides) specific to *Sutherlandia frutescens* extracts

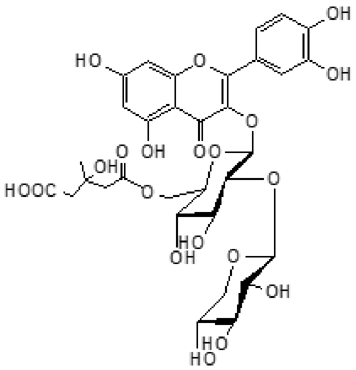

Sutherlandin A (Quercetin)

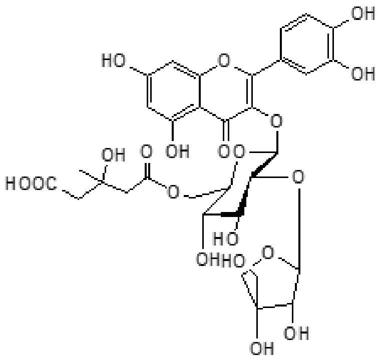

Sutherlandin B

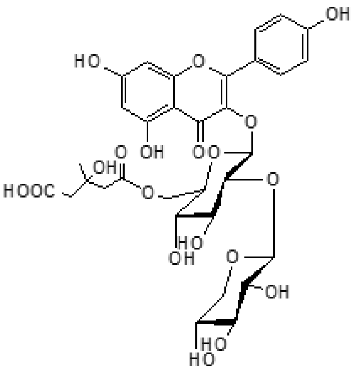

Sutherlandin C (Kaempferol)

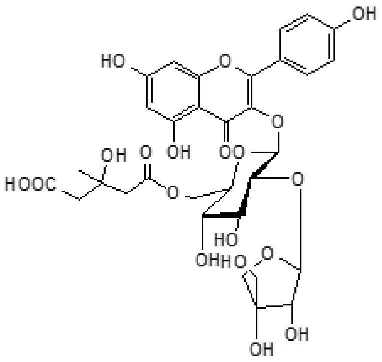

Sutherlandin D

Figure S2. Structures of flavonoids specific (sutherlandins) to *Sutherlandia frutescens* extracts.

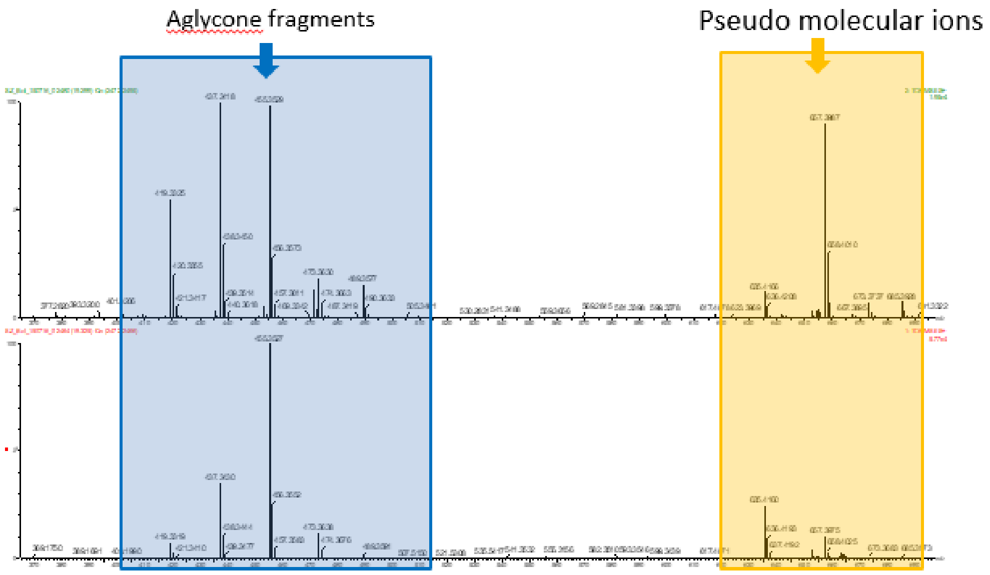

Figure S3. Example MS spectra showing the low energy scan below and the high energy scan above, with the typical sutherlandioside aglycone fragments, m/z 455, 437, 419, 473 and 489 and the M+H (m/z 653), and, M+Na (m/z 657) ions.
